# Metaflammasome components in the human brain: a role in dementia with Alzheimer's pathology?

**DOI:** 10.1111/bpa.12388

**Published:** 2016-06-08

**Authors:** Mariko Taga, Thais Minett, John Classey, Fiona E. Matthews, Carol Brayne, Paul G. Ince, James AR Nicoll, Jacques Hugon, Delphine Boche, MRC CFAS

**Affiliations:** ^1^ Clinical Neurosciences, Clinical and Experimental Sciences Academic Unit, Faculty of Medicine University of Southampton Southampton, UK; ^2^ INSERM U942, Paris France; ^3^ Department of Public Health and Primary Care Institute of Public Health, University of Cambridge Cambridge, UK; ^4^ Department of Radiology University of Cambridge Cambridge, UK; ^5^ MRC Biostatistics Unit Cambridge Institute of Public Health Cambridge, UK; ^6^ Department of Neuroscience, Sheffield Institute for Translational Neuroscience, Sheffield University Sheffield, UK; ^7^ Department of Cellular Pathology University Hospital Southampton NHS Foundation Trust Southampton UK; ^8^ University of Paris Diderot Sorbonne Paris Cité Paris France; ^9^ Centre Memoire de Ressources et de Recherche Paris Nord Ile de France AP‐HP Hôpital Lariboisière Paris France

**Keywords:** dementia, metaflammasome, CFAS, Alzheimer's disease, human brain

## Abstract

Epidemiological and genetic studies have identified metabolic disorders and inflammation as risk factors for Alzheimer's disease (AD). Evidence in obesity and type‐2 diabetes suggests a role for a metabolic inflammasome (“metaflammasome”) in mediating chronic inflammation in peripheral organs implicating IKKβ (inhibitor of nuclear factor kappa‐B kinase subunit beta), IRS1 (insulin receptor substrate 1), JNK (c‐jun N‐terminal kinase), and PKR (double‐stranded RNA protein kinase). We hypothesized that these proteins are expressed in the brain in response to metabolic risk factors in AD. Neocortex from 299 participants from the MRC Cognitive Function and Ageing Studies was analysed by immunohistochemistry for the expression of the phosphorylated (active) form of IKKβ [pSer^176/180^], IRS1 [pS^312^], JNK [pThr^183^/Tyr^185^] and PKR [pT^451^]. The data were analyzed to investigate whether the proteins were expressed together and in relation with metabolic disorders, dementia, Alzheimer's pathology and *APOE* genotype. We observed a change from a positive to a negative association between the proteins and hypertension according to the dementia status. Type‐2 diabetes was negatively related with the proteins among participants without dementia; whereas participants with dementia and AD pathology showed a positive association with JNK. A significant association between IKKβ and JNK in participants with dementia and AD pathology was observed, but not in those without dementia. Otherwise, weak to moderate associations were observed among the protein loads. The presence of dementia was significantly associated with JNK and negatively associated with IKKβ and IRS1. Cognitive scores showed a significant positive relationship with IKKβ and a negative with IRS1, JNK and PKR. The proteins were significantly associated with pathology in Alzheimer's participants with the relationship being inverse or not significant in participants without dementia. Expression of the proteins was not related to *APOE* genotype. These findings highlight a role for these proteins in AD pathophysiology but not necessarily as a complex.

## Introduction

The number of people living with dementia is estimated at 35.6 million persons with the number to double by 2030 and triple by 2050, costing already $604 billion in 2010 according the WHO and the Alzheimer's Disease International 1. The major cause of dementia is Alzheimer's disease (AD) which is responsible for about two thirds of cases. AD pathology is characterised by the accumulation of amyloid‐β peptide (Aβ) plaques, hyperphosphorylated tau protein within neuronal somata and processes, neuroinflammation and neuronal loss. However, Aβ and to a lesser extent tau accumulation are also frequently observed in the brains of non‐demented persons 2. This suggests that key features of AD are neither necessary nor sufficient for the development of cognitive failure, even in the absence of other dementia‐associated pathology. In contrast, systemic inflammation 3 and tangle pathology 4 correlate closely with cognitive decline, but the underlying biological mechanisms are poorly understood. The heritability of AD is between 0.6 and 0.7 5, 6, 7, and a long established genetic risk factor for AD is polymorphism of the apolipoprotein E (*APOE*) gene 8, 9. More recently, large‐scale genome‐wide association studies (GWAS) have clearly implicated genetic variation in innate immunity and other aspects of lipid metabolism 10, 11, 12. In addition, several environmental risk factors for AD currently of great importance in the field of public health include type 2 diabetes 13, 14, 15, obesity 16, 17, 18, midlife hypertension 19, 20, 21 and systemic infection 22. Interestingly, these environmental risk factors are all associated with disorders of lipid metabolism and/or the induction of chronic low‐grade systemic inflammation 23, 24, 25.

The inflammasome is a multiprotein complex expressed in myeloid cells and a component of the innate immune system responsible for activation of inflammatory processes 26. Experimental studies implicate involvement of the inflammasome in the initiation or progression of diseases with an impact on public health, such as metabolic disorders and neurodegenerative diseases 27. Based on the concept of the inflammasome, a metabolic inflammasome or “metaflammasome” has been introduced to describe the cellular signaling reaction observed after stress induced by misfolded protein in the endoplasmic reticulum, lipid stress (eg, obesity, type 2 diabetes) or infection 28, linking together the metabolic disorder and inflammation observed in periphery 29. Experimental studies have identified the kinases IKKβ (inhibitor of nuclear factor kappa‐B kinase subunit beta), IRS1 (insulin receptor substrate 1), JNK (c‐jun N‐terminal kinase) and PKR (double‐stranded RNA protein kinase) as major contributors to the induction of inflammation in tissue affected by metabolic disorders 28, 29, 30. In response to nutrient or inflammatory signals, PKR becomes activated, leading to the phosphorylation of JNK, IKKβ and IRS1, resulting in inhibition of the insulin receptor signalling cascade 28, 30. PKR and JNK are also known to be involved in human AD with elevated PKR and JNK detected in the cerebrospinal fluid (CSF) of AD patients and their levels associated with the rate of cognitive decline 31, 32.

In this study, we hypothesize that these four proteins are expressed in the brain together in response to metabolic risk factors and are analogous to the metaflammasome complex described in the periphery. We also tested whether the phosphorylated (active) forms of these proteins were associated with (i) dementia status; (ii) cognitive impairment; (iii) specific features of AD pathology and (iv) *APOE* genotype. We have explored these hypotheses in the large well‐studied MRC Cognitive Function and Ageing Study (CFAS) *postmortem* cohort.

## Materials and Methods

### The CFAS cohort

The CFAS study involves six centres in the UK (Liverpool, Cambridge, Gwynedd, Newcastle, Nottingham and Oxford). The design and methods have been described in detail elsewhere 33. In brief, the project began in the early 1990s and recruited individuals aged 65 years and over living in the community. The main aims were to estimate the prevalence and incidence of cognitive decline and dementia; to determine the rate of progression of cognitive decline and survival, and to identify risk factors for cognitive decline and dementia. Baseline prevalence screening of the cohort included sociodemographic, cognitive, physical health and medication data. Participants were asked to consent to brain donation after death. The ascertainment of dementia status at death has already been published 34 and was based on review of information available from death certificate, last interview assessment and the informants' information about participants' function and cognition (mini mental state examination—MMSE—score) during the last years of life. A total of 299 brains were used in this study with the demographic and cognitive profile of the cohort described in Table 1. In 21 cases, insufficient information was available for a diagnosis of dementia to be made; these cases are included in pathological analyses but excluded from the study of dementia interactions. In 15 cases, there were no available data regarding hypertension or type 2 diabetes. Among those with available information, 42% of the participants had hypertension and 12% had type 2 diabetes, both self‐reported either by the participant, a relative or the career. The study received ethical approval from the Cambridgeshire 1 Research Ethics Committee (Rec number: 10/H0304/61/).

**Table 1:** Characteristics of the cohort according to dementia status and metaflammasome components.

	No dementia (n = 130)	Overall dementia (n = 148)	Dementia with AD pathology (n = 83)
Number of women (%)	66 (51)	102 (69)	53 (64)
Age at death^†^	84 (77–90)	89 (84–93)	89 (83–93)
Years since last cognitive assessment^†^	1.1 (0.5–1.8)	1.7 (0.8–3.1)	1.5 (0.8–3.2)
MMSE at last assessment^†^	25 (22–28)	14 (8–20)	11 (6–17)
IKKβ load (%)^††^	0.302 (0.002)	0.282 (0.002)	0.279 (0.003)
IRS1 load (%)^††^	0.391 (0.003)	0.379 (0.003)	0.360 (0.003)
JNK load (%)^††^	0.266 (0.005)	0.274 (0.003)	0.302 (0.004)
PKR load (%)^††^	0.542 (0.005)	0.540 (0.005)	0.551 (0.006)

^†^Median, inter quartile range.

^††^Linearized mean (linearized standard error) expressed as protein load (%).

### Assessment of Alzheimer pathology

Pathological evaluation of the CFAS cohort has been previously described 34 and was conducted by neuropathologists, blind to clinical data, using immunohistochemical or tinctorial methods. The severity of diffuse plaques, neuritic plaques and tangles was scored semiquantitatively according to the Consortium to Establish a Registry for Alzheimer's disease (CERAD) protocol as either “none,” “mild,” “moderate” or “severe.” For the analysis, the scores were simplified as the score “severe” did not occur very often. It was merged with “moderate,” and the score “mild” was merged with “none.” Cerebral amyloid angiopathy (CAA) was assessed in the meninges and parenchyma on a similar semiquantitative scale. At the end of the assessment a final neuropathological diagnosis of AD based on the distribution and severity of plaques and tangles, but blind to any clinical information was made.

### Immunohistochemistry

**Figure 1:**
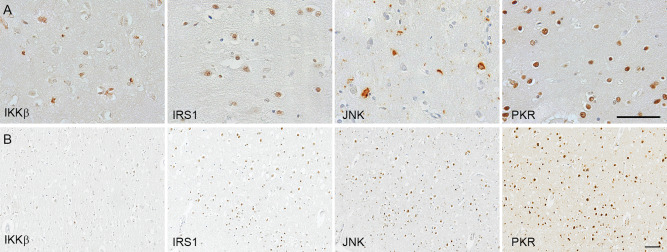
A. Illustration of immunostaining of the metaflammasome phosphorylated components: IKKβ [pSer^176/180^] (inhibitor of nuclear factor kappa‐B kinase subunit beta), IRS1 [pS^312^] (Insulin Receptor Substrate 1), JNK [pThr^183^/Tyr^185^] (c‐jun N‐terminal kinase), and PKR [pT^451^] (Double‐stranded RNA protein kinase) in human *post‐mortem* brain. **B.** Illustration of the number of cell‐positive for each protein in a participant with dementia and AD pathology. Haematoxylin counterstaining; scale bar: (A) 20μm; (B) 100μm

The following primary antibodies directed against the phosphorylated (active) form were used: rabbit anti‐IKKβ [pSer^176/180^] (Cell Signaling, Hertfordshire, UK); rabbit anti‐IRS1 [pS^312^] (Invitrogen, Loughborough UK); rabbit anti‐JNK [pThr^183^/Tyr^185^] (Cell Signaling, Hertfordshire UK) and rabbit anti‐PKR [pT^451^] (Invitrogen, Loughborough UK) (Figure 1).

Four micrometer sections of formalin‐fixed paraffin‐embedded tissue from the middle frontal gyrus, a region which is part of the CERAD neuropathology assessment for the diagnosis of AD, were used for immunostaining for microglial proteins. Immunohistochemistry was performed using the appropriate antigen retrieval methods for each primary antibody. Biotinylated secondary antibodies were from Dako (Glostrup, Denmark), and normal serum and avidin–biotin complex from Vector Laboratories (Peterborough, UK). Biotinylated antibody was visualized using the avidin–biotin‐peroxidase complex method [Vectastain Elite ABC from Vector Laboratories (Peterborough, UK)] with 3,3′ diaminobenzidine [DAB, Vector Laboratories (Peterborough, UK)] as chromogen and 0.05% hydrogen peroxide as substrate. All sections were counterstained with haematoxylin then dehydrated before mounting in DePeX (VWR International, Lutterworth, UK). Cases were immunolabeled together in batches to ensure compatibility of staining and sections incubated in the absence of the primary antibody were included as negative controls. For each antibody, a positive control was included to ensure staining consistency across the different batch runs.

### Quantification

Quantification was performed blind to the experimental group and identity of the cases. Images of the slides were taken from the same anatomical regions in every case as identified by a neuropathologist (JN). For each antibody, 30 images of cortical grey matter at magnification ×20 were taken per case in a zigzag sequence along the cortical ribbon to ensure that all cortical layers were represented in the quantification in an unbiased manner. The acquired images were analyzed using ImageJ (version 1.49m, Wayne Rasband, NIH, USA), with a threshold applied to the image to select and measure the total amount of specific immunostaining. The same threshold setting was maintained to all images of all cases stained for the same antibody and the area fraction of the measure function provided the proportion (%) of the stained area related to the total area of the image (expressed as protein load) 35. A macro was designed to incorporate all the steps allowing automatic image processing and data collection. The data were then sent to the Department of Public Health and Primary Care for statistical analysis.

### Statistical analysis

The analyses were performed by Dr Minett and Prof Matthews, Professor of Epidemiology and Principal Statistician on the CFAS. Means (standard deviation) or median (interquartile range) are reported. The Spearman correlation coefficients were calculated to verify the strength of the relationship between IKKβ, IRS1, JNK and PKR expressions.

Their relationships with dementia status and frontal lobe neurodegenerative pathologies were verified using weighted logistic regression where the 30 images acquired for each microglial protein were given the same 1/30 weight.

Weighted multiple linear regression analysis to assess whether metaflammasome protein expression was related to hypertension, type 2 diabetes and cognition (MMSE) after adjustment for the interval between last interview and death.

To verify the association of *APOE* genotype with metaflammasome protein expression (dependent variables), weighted logistic regressions were performed with ε2 and ε4 carrier status used as independent variables regardless of the number of alleles, and with both alleles simultaneously present in the analysis. Sensitivity analyses were performed to verify if the relationships predicted by the regressions were stable. For this, the metaflammasome component loads were divided into quartiles and the categorised data used rather than the raw scores into the weighted regression analyses.

All regression analyses were adjusted for age of death and sex. All tests were 2‐tailed and statistical analyses were performed using the statistical package STATA, version 12. A *P*‐value < 0.05 was considered as significant, unless a potential problem of multiple comparisons was identified, in which case, the critical *P*‐values were adjusted according to the Bonferroni's method.

## Results

### Characteristics of the cohort regarding dementia status

Among the 299 participants, 148 (69%) cases had dementia at death (Table 1) and for 21 (7%) cases the dementia status was unknown. Of the 148 cases with dementia, 83 (56%) had plaques and tangles sufficient for the diagnosis of AD as the cause of dementia. For the participants without dementia, 66 (51%) were women, the median age at death was 84 years (77–90) and the median MMSE score performed at the last assessment was 25 22, 23, 24, 25, 26, 27, 28. For the group with dementia, 102 (69%) were women, including 64% with AD pathology, with median age at death of 89 years (83–93). The median MMSE score performed at the last assessment for the dementia group was 14 8, 9, 10, 11, 12, 13, 14, 15, 16, 17, 18, 19, 20 and 11 6, 7, 8, 9, 10, 11, 12, 13, 14, 15, 16, 17 for people with dementia with AD pathology (Table 1).

### Immunodetection of the metaflammasome components in the brain

All four components of the metaflammasome: IKKβ, IRS1, JNK and PKR were detected by immunohistochemistry in neurons in the cerebral cortex as illustrated in Figure 1A. For PKR and IRS1, the staining was nuclear, cytoplasmic for IKKβ, and present in both neuronal compartments for JNK, as expected from the literature. The immunodetection shows a difference in the cell density between the different metaflammasome proteins as illustrated in the brain of a participant with dementia and AD pathology (Figure 1B). Interestingly, some of the participants without dementia did not expressed JNK. The quantified protein load (%) for each protein used for the analyses is presented in Table 1.

### Metaflammasome proteins and metabolic risk factors for AD

We assessed the association of the expression of metaflammasome proteins and two metabolic disorders known as risk factors for AD: hypertension and type 2 diabetes.

Among participants without dementia, a significant positive relationship was detected between hypertension and IKKβ, IRS1 and JNK. In contrast, among participants with dementia and AD pathology, all components of the metaflammasome were negatively associated with hypertension (Table 2). Type 2 diabetes was negatively associated with IKKβ, IRS1 and JNK among participants without dementia; whereas among participants with dementia and AD pathology, a significant negative association was observed with IRS1 and PKR, and a significant positive relationship with JNK (Table 2).

**Table 2:** Weighted multiple linear regression to investigate the relationship between metaflammasome components and hypertension and self‐reported type 2 diabetes.

Metaflammasome components [load (%)]	No Dementia			Dementia with AD pathology		
	*β*	95%CI(β)	*P*	*β*	95%CI(β)	*P*
Hypertension						
IKKβ	0.02	(0.02; 0.03)	<0.001	−0.03	(−0.05; −0.02)	<0.001
IRS1	0.04	(0.03; 0.06)	<0.001	−0.03	(−0.04; −0.01)	0.006
JNK	0.07	(0.05; 0.09)	<0.001	−0.05	(−0.07; −0.03)	<0.001
PKR	−0.01	(−0.03; 0.01)	0.281	−0.09	(−0.11; −0.06)	<0.001
Type 2 diabetes						
IKKβ	−0.02	(−0.03; −0.01)	0.004	0.00	(−0.02; 0.02)	0.793
IRS1	−0.05	(−0.06; −0.03)	<0.001	−0.05	(−0.07; −0.02)	<0.001
JNK	−0.05	(−0.07; −0.04)	<0.001	0.10	(0.06; 0.13)	<0.001
PKR	−0.01	(−0.04; 0.01)	0.225	−0.07	(−0.11; −0.02)	0.005

*P*‐value < 0.012 considered to indicate statistical significance according to the Bonferroni's method.

Significant positive association (dark gray); Significant negative association (light gray).

### Interaction of the metaflammasome proteins

**Figure 2:**
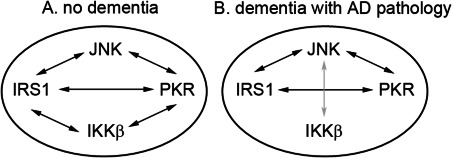
Cartoon to illustrate the interactions of the different metaflammasome phosphorylated proteins in the human brain in relation with the dementia status. **A.** In the absence of dementia, positive associations (black arrows) are observed between JNK, IRS1 and PKR and between IKKβ, IRS1 and PKR. No relationship is observed between JNK and IKKβ. **B.** In the presence of dementia with Alzheimer's pathology, relationship between IKKβ with IRS1 and PKR are lost and an inverse association (gray arrow) is formed between JNK and IKKβ.

The overall correlations between the metaflammasome proteins were weak or moderate. Among participants without dementia, significant positive correlations were detected except between IKKβ and JNK. Among participants with dementia and AD pathology, significant positive correlations were observed between JNK with PKR and IRS1, and PKR with IRS1. A negative correlation was detected between IKKβ and JNK. There were no significant correlations between IKKβ with IRS1 and PKR (Table 3 and Figure 2).

**Table 3:** Correlation matrix of the metaflammasome components [load (%)].

Correlation	No dementia		Dementia with AD pathology	
	*r*	*P*	*r*	*P*
IKKβ × IRS1	0.33	<0.001	0.15	0.171
IKKβ × JNK	0.07	0.463	−0.29	0.009
IKKβ × PKR	0.25	0.004	−0.13	0.260
IRS1 × JNK	0.43	<0.001	0.30	0.006
IRS1 × PKR	0.34	<0.001	0.38	<0.001
JNK × PKR	0.59	<0.001	0.66	<0.001

*P*‐value < 0.008 considered to indicate statistical significance according to the Bonferroni's method.

Significant positive association (dark gray); Significant negative association (light gray).

### Metaflammasome proteins, dementia status and MMSE scores

Dementia status (ie, dementia vs. no dementia) and MMSE scores were analyzed in relation to the metaflammasome proteins. First for dementia status, we observed in the whole cohort after sensitivity analysis, a significant negative relationship with IKKβ, indicating that the participants with low expression of IKKβ were likely to have dementia. The analysis in participants with dementia and AD pathology revealed in addition to the significant negative relationship with IKKβ, a significant negative association with IRS1 and a significant positive relationship with JNK. Thus, participants with dementia and AD pathology are likely to have expression of relatively higher levels of JNK and lower levels of IKKβ and IRS1 relative to participants without dementia (Table 4).

**Table 4:** Weighted logistic regression to analyse the relationship between metaflammasome components and dementia.

Metaflammasome components [load (%)]	OR	95%CI(OR)	*P*
Dementia/no Dementia			
IKKβ^†^	0.2	(0.2; 0.3)	<0.001
IRS1	0.5	(0.4; 0.7)	<0.001
JNK	1.1	(0.9; 1.3)	0.338
PKR	0.9	(0.8; 1.0)	0.059
Dementia with AD pathology/no Dementia			
IKKβ^†^	0.2	(0.1; 0.4)	<0.001
IRS1^†^	0.3	(0.2; 0.4)	<0.001
JNK^†^	1.5	(1.2; 1.9)	<0.001
PKR	1.0	(0.8; 1.1)	0.617

*P*‐value < 0.012 considered to indicate statistical significance according to the Bonferroni's method.

^†^Significant positive association (dark gray) and significant negative association (light gray) with the relationship maintained in the sensitivity analysis.

Second, in relation to the MMSE score, we showed among the whole cohort a significant positive relationship with IKKβ and a significant negative relationship with IRS1, JNK and PKR. Therefore, good cognition was associated with higher IKKβ expression and lower IRS1, JNK and PKR expression (Table 5).

**Table 5:** Weighted multiple linear regression to analyse the relationship between metaflammasome components and the MMSE score.

Metaflammasome components [load (%)]	*β*	95%CI(β)	*P*
IKKβ	4.0	(2.8; 5.3)	<0.001
IRS1	−1.3	(−2.0; −0.6)	<0.001
JNK	−2.2	(−2.7; −1.6)	<0.001
PKR	−0.7	(−1.1; −0.3)	0.001

*P*‐value < 0.012 considered to indicate statistical significance according to the Bonferroni's method.

Significant positive association (dark gray); Significant negative association (light gray).

### Metaflammasome proteins and AD neuropathology

Among participants without dementia, the significant relationships observed between metaflammasome proteins and AD neuropathology were negative between: IKKβ and meningeal CAA; IRS1 and diffuse and neuritic plaques; and JNK and neuritic plaques (Table 6). The other relationships were not maintained in the sensitivity analysis.

**Table 6:** Weighted logistic regression to investigate the relationship between metaflammasome components and Alzheimer's pathology according to disease status.

Metaflammasome components [load (%)]	Meningeal CAA	Parenchymal CAA	Diffuse plaques	Neuritic plaques	Tangles
	OR [95%CI(OR)]*P*	OR [95%CI(OR)]*P*	OR [95%CI(OR)]*P*	OR [95%CI(OR)]*P*	OR [95%CI(OR)]*P*
No Dementia					
IKKβ	0.00 (0.00; 0.02) <0.001^†^	0.00 (0.00; 0.00) <0.001	0.86 (0.51; 1.45) 0.573	2.65 (1.33; 5.31) 0.006	391.08 (135.07; 1132.32) <0.001
IRS1	0.04 (0.02; 0.09) <0.001	0.00 (0.00; 0.00) <0.001	0.22 (0.15; 0.31) <0.001^†^	0.49 (0.31; 0.79) 0.003^†^	0.77 (0.19; 3.16) 0.714
JNK	0.11 (0.06; 0.21) <0.001	0.28 (0.14; 0.56) <0.001	1.07 (0.88; 1.31) 0.473	0.60 (0.44; 0.81) 0.001^†^	0.00 (0.00; 0.02) <0.001
PKR	0.25 (0.14; 0.44) <0.001	1.40 (0.97; 2.03) 0.072	0.76 (0.62; 0.93) 0.008	1.10 (0.84; 1.44) 0.502	0.08 (0.05; 0.14) <0.001
Dementia with AD pathology					
IKKβ	0.51 (0.26; 0.98) 0.043	1.62 (0.72; 3.64) 0.242	3.89 (1.54; 9.79) 0.004^†^	70.24 (27.06; 182.30) <0.001^†^	5.26 (2.92; 9.48) <0.001
IRS1	0.79 (0.45; 1.38) 0.403	1.21 (0.64; 2.30) 0.555	1.66 (0.93; 2.95) 0.087	5.07 (2.75; 9.34) <0.001	1.34 (0.81; 2.21) 0.260
JNK	8.98 (5.66; 14.25) <0.001^†^	10.93 (6.46; 18.47) <0.001^†^	3.11 (1.95; 4.96) <0.001	5.96 (3.94; 9.01) <0.001^†^	1.09 (0.74; 1.61) 0.650
PKR	3.63 (2.74; 4.83) <0.001	7.35 (5.22; 10.35) <0.001^†^	4.23 (3.08; 5.82) <0.001	1.95 (1.52; 2.49) <0.001	1.68 (1.27; 2.21) <0.001

*P*‐value < 0.012 considered to indicate statistical significance according to the Bonferroni's method.

^†^Significant positive association (dark gray) and significant negative association (light gray) with the relationship maintained in the sensitivity analysis.

In the participants with dementia and Alzheimer's pathology, the significant maintained relationships were mainly positive and stronger than those in the participants without dementia. IKKβ expression was significantly related to diffuse and neuritic plaques. JNK was strongly related with meningeal and parenchymal CAA and neuritic plaques, and PKR with parenchymal CAA (Table 6). The other associations were not maintained in the sensitivity analysis.

### Metaflammasome proteins and APOE polymorphism

With regards to the *APOE* polymorphism, the expression of metaflammasome proteins did not change the dementia risk conferred by the *APOE* genotype (data not shown).

## Discussion

Our data show that the four kinases (IKKβ, IRS1, JNK and PKR) are all expressed in the human brain. However, they might not act as a metaflammasome complex as hypothesized in experimental metabolic disease 30, due to the absence of some associations, the weakness of the observed correlations and the difference in the cell density expressing the proteins. Nevertheless our findings highlight a role for these proteins in association with metabolic disorders (eg, hypertension and type 2 diabetes), dementia and to a lesser extent with AD pathology.

It is essential to note that the major value of studying the human brain in this way is that it is the study of the disease itself rather than an experimental model of some aspect of the disease in the absence of the usual comorbidities observed in the aged population and which does not inform specifically on human AD. However, this approach also has some limitations which have to be considered. First, inherent to the use of postmortem tissue, the study is by nature observational and cannot demonstrate cause and effect or any directionality. Therefore, we have investigated associations but not mechanisms of these proteins in the context of dementia with the neuropathological hallmarks of AD. Our study is based on the hypothesis of the presence of a putative metaflammasome in relation with inflammation and risk factors for AD, as suggested in the periphery 29 in experimental studies 28. Second, our analysis of postmortem tissue may not necessarily reflect the earliest effects of the metaflammasome proteins in dementia, hypertension or diabetes but mainly the end stage of the disease; although an advantage of the population‐based CFAS approach is that it includes the full spectrum of cognition from unimpaired to frank dementia.

Other limitations include that the information on hypertension and diabetes is self‐reported to CFAS by the participants or carers rather than obtained from the medical records, and the potentially confounding effects of medication taken in relation to the above comorbidities. To mitigate these limitations, the discussion of the findings has been based on the sustained significance after sensitivity analysis or adjusted multiple comparisons as an indication of the robustness of the relationship.

Our first hypothesis was that the metaflammasome proteins were expressed in the human brain in association with metabolic disorders and AD. Immunodetection confirmed the presence in the human brain of the four components of the metaflammasome complex (ie, the phosphorylated forms of IKKβ, IRS1, JNK and PKR). Both PKR and IRS1 locations were nuclear, consistent with previous detection in AD brain 36, 37. IKKβ was detected in the cytoplasm of neurons consistent with previous studies showing a cytoplasmic location 38. JNK showed either a nuclear or a cytoplasmic location as already published, potentially underlying distinct functions of JNK based on the cell compartment 39.

The concept of the metaflammasome, analogous to the inflammasome, was suggested in metabolic disease. Therefore, we wanted to know whether the expression of these four proteins was associated with metabolic disorders. Data concerning two important metabolic disorders known as risk factors for AD were available from the CFAS database, hypertension and type 2 diabetes. Analysis with regard to hypertension showed an association with the metaflammasome proteins with a change in the relationship pattern according to the dementia status. A significant positive association was detected in the participants without dementia and an inverse association in participants with dementia and AD pathology. Several studies have revealed a positive association between high blood pressure in midlife (40–64 years old) and the development of dementia 40, 41, 42, 43, whereas only two studies supported an association between hypertension in late‐life and dementia 44, 45. Indeed, several studies suggested that in old age, hypotension could be a higher risk factor for AD, after adjustment for antihypertensive drugs 45, 46, 47, 48. The working hypothesis is that long‐standing hypertension may lead to severe atherosclerosis/arteriolosclerosis and impaired cerebrovascular autoregulation, whereas a decline in blood pressure in later life may contribute to diminished cerebral perfusion leading to an ischaemic state and increased cerebral Aβ accumulation 42. Our findings in the CFAS cohort support this hypothesis, that participants without dementia are more likely than participants with dementia to have elevated high blood pressure.

Regarding type 2 diabetes, significant inverse associations were found with the metaflammasome proteins in both cohorts, except for JNK. Indeed for JNK, the relationship was significantly inverse in participants without dementia and positive in participants with dementia. Our data imply that in participants with type 2 diabetes, a low expression of JNK is more likely to be associated with the absence of dementia; whereas a high expression will be preferably observed in participants with dementia and AD pathology. Experimental studies have shown that: (i) inhibition of JNK in the liver leads to a beneficial effect on insulin resistance and glucose tolerance 49; (ii) JNK is abnormally activated in obesity 28 and (iii) its absence leads to a decrease in adiposity and to an improvement in insulin sensitivity 50. These findings and our observation in human *postmortem* tissue indicates that JNK could change its function in type 2 diabetes in the context of dementia.

A matrix analysis was performed to investigate whether the four proteins were acting together as a putative complex 30. The associations detected between the different components were either weak or moderate. Interestingly, differences in the associations were observed according to the dementia status. In the absence of dementia, there was no association between IKKβ and JNK; whereas in the context of AD, a significant inverse relationship was reported between IKKβ and JNK with the loss of relationship of IKKβ with IRS1 and PKR as illustrated in Figure 2. These findings suggest that the relationship between IKKβ and other components of the metaflammasome is unstable and potentially might change according the brain environment. Although, our data are not consistent with the concept of a metaflammasome complex as described in the periphery, they do nevertheless support a role of these proteins in AD. Second, the role of these proteins in AD might be driven by the relationship between IKKβ and JNK.

This finding is consistent with the observation that dementia status and poor cognition were significantly related to the expression of JNK, whereas the presence of IKKβ was related to good cognitive function and the absence of dementia. This is in accordance with a recent study which demonstrated an increased level of JNK in the CSF of 30 AD patients associated with the rate of their cognitive decline 32. Interestingly the link between dementia and JNK appears only among the participants with dementia and AD pathology while the relationship was not observed within the whole cohort of participants or within participants with non‐AD dementia. This supports the hypothesis for a specific role for JNK in AD pathogenesis, rather than JNK being a marker of the pathological processes associated with any form of dementia. IKKβ plays a role in the coordination of the inflammatory responses through activation of the NF‐κB pathway 51. Its neuroprotective function, as suggested by our study, supports previous *in vitro* findings with the expression of neuronal NF‐κB protecting against Aβ toxicity 52 and oxidative stress 53, and the inhibition of neuronal NF‐κB resulting in the loss of neuroprotection 54. Interestingly, with regards to the two other proteins involved in the putative metaflammasome, IRS1 and PKR, we observed a high expression related with worse cognition, but not with the presence of dementia (frank dementia usually reflecting the latest stage of neurodegeneration), perhaps implying that these components might be involved in the earlier stages of AD or in mild cognitive impairment. This is supported by a recent study showing that mice developing insulin resistance and increased hippocampal IRS1 following a high fat diet had a deficiency of spatial working memory due to postsynaptic impairment 55, the most reliable index of cognition as observed in *postmortem* and biopsy studies of AD brain 56. PKR is a pro‐apoptotic serine/threonine kinase, the activation of which by phosphorylation initiates a cascade of neurodegenerative cellular events leading to apoptosis 57. Assay of PKR in CSF has been shown to predict cognitive decline 31, 58 supporting the idea of PKR as a biomarker for AD 59.

In relation to AD pathology, among the participants without dementia, there was either no association or a significant inverse association between the metaflammasome proteins and AD neuropathology. In contrast, in the participants with dementia and AD pathology, the metaflammasome proteins were associated with AD pathology, especially strongly for IKKβ and plaques, and for JNK with CAA and neuritic plaques. The hypothesis of a metaflammasome is based on the concept that failure of endoplasmic reticulum function (due to accumulation of newly synthesized unfolded proteins) results in the activation of an unfolded protein response and upregulated inflammation 30. Our data in part appear to support this concept as in AD the accumulation of Aβ and Tau might lead to an unfolded protein response and inflammation, hence precipitating neurodegeneration. Interestingly, IRS1 was inversely associated with plaques in the participants without dementia and showed no significant relationship with AD pathology in the participants with dementia and AD pathology, therefore re‐enforcing the idea of an early role of IRS1 in AD pathogenesis. The only observed significant association maintained after the sensitivity analysis between PKR and AD pathology was with parenchymal CAA in the participants with dementia and AD pathology. In AD, PKR partially colocalises with phosphorylated tau 36, and thus, identifies neurons susceptible to neurodegeneration and is generally considered as a marker of early neurodegeneration 60. The relationship between PKR and CAA might reflect neuronal death associated with Aβ accumulation in the vasculature. IKKβ is associated with good cognition and its relationship with plaques in the participants with dementia and AD pathology raises the question of the neurotoxicity of fibrillary Aβ 61, 62. It is important to note that in the participants without dementia, the relationship between IKKβ and tangles was not sustained after the sensitivity analysis, despite the high odds ratio (391), due to the extremely low prevalence of tangles in this cohort. The association of JNK with CAA and neuritic plaques reflects that both features are associated with neurodegeneration, cognitive decline and dementia 63. Recently, increased JNK expression was observed in the brains of AD patients associated with amyloid pathology 32. Overall, the findings support the hypothesis that the cerebral metaflammasome proteins might be involved in AD pathogenesis; mainly in relation to the Aβ peptide rather than tau protein, as the significant relationships observed with tangles were not maintained in the sensitivity analysis.

Interestingly, the expression of the components of the metaflammasome is independent of the *APOE* gene polymorphism, the main risk factor for AD 64.

Overall, our study supports a role for these four kinases in the human AD brain, but the formation of a metaflammasome complex as suggested by the experimental studies remains questionable. Intriguingly, the effect appears to be led by the relationship between IKKβ and JNK. This could explain the difference observed in the expression of the metaflammasome regarding dementia, cognition and hypertension. This supports previous studies in which a pro‐survival mechanism of the NF‐κB pathway, by suppressing apoptosis through the inhibition of JNK signaling was described 65, 66, 67, with the IKKβ/NF‐κB pathway and JNK signaling leading to opposite roles during apoptosis with the antiapoptotic function mediated through the attenuation of JNK activity 68. Our data in the human brain support the exploration of the use of inhibitors of kinase, such as JNK inhibitors, currently developed for B cell‐related haematological cancers as potential drugs for AD.

## Declaration of Interest

The authors do not have conflict of interest.
